# Analysis of Amygdalin in Various Matrices Using Electrospray Ionization and Flowing Atmospheric-Pressure Afterglow Mass Spectrometry

**DOI:** 10.3390/biom10101459

**Published:** 2020-10-19

**Authors:** Maria Guć, Sandra Rutecka, Grzegorz Schroeder

**Affiliations:** Faculty of Chemistry, Adam Mickiewicz University in Poznan, Uniwersytetu Poznańskiego 8, 61-614 Poznań, Poland; san-rut@wp.pl (S.R.); schroede@amu.edu.pl (G.S.)

**Keywords:** amygdalin, mag-MIP, ESI-MS, FAPA-MS

## Abstract

Amygdalin is a natural cyanogenic compound that plants produce in the fight against insects and herbivores. Excessive amounts of amygdalin by animals and humans can potentially lead to fatal intoxication. However, studies confirm that amygdalin has antitumor properties, including the ability to inhibit the proliferation of cancer cells and to induce their apoptosis. The analysis of amygdalin in various matrices is an important analytical problem today. The publication presents the methodology of direct determination of amygdalin in water, sewage, and biological materials using electrospray ionization mass spectrometry (ESI-MS) and a new analytical method using flowing atmospheric-pressure afterglow mass spectrometry (FAPA-MS). The methods of analyte pre-concentration using a magnetic, molecularly imprinted polymer (mag-MIP) and the influence of interferents on the recorded spectra were discussed. Analytical parameters in ESI-MS and FAPA-MS methods were established. The linearity range was 4.5 µg L^−1^–45 mg L^−1^ in positive mode ESI-MS and FAPA-MS. The limit of detection (LOD) for ESI-MS was 0.101 ± 0.003 µg L^−1^ and the limit of quantification (LOQ) was 0.303 ± 0.009 µg L^−1^. In FAPA-MS, the LOD was 0.050 ± 0.002 µg L^−1^ and the LOQ was 0.150 ± 0.006 µg L^−1^. The content of amygdalin in various matrices was determined.

## 1. Introduction

**Figure 1:**
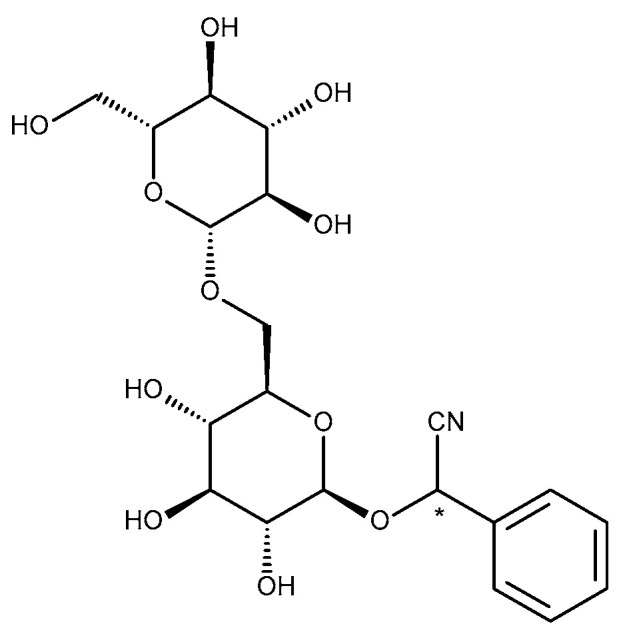
Amygdalin structural formula.

Amygdalin is a natural chemical compound of plant origin, belonging to the group of cyanogenic glycosides. Natural amygdalin has the (*R*)-configuration at the chiral phenyl center. Under mild basic conditions, this stereogenic center isomerizes; the (*S*)-epimer is called neoamygdalin. Although the synthesized version of amygdalin is the (*R*)-epimer, the stereogenic center attached to the nitrile and phenyl groups easily epimerizes (Figure 1) [1,2,3].

Amygdalin is found in plants belonging to the Rosaceae and the Passifloraceae families. Its harmfulness is associated with hydrogen cyanide released in the metabolic process. This provides a natural defense system. Amygdalin taken orally is highly toxic. Hydrogen cyanide released as a result of enzymatic hydrolysis of amygdalin causes nausea, headaches and dizziness, convulsions, and even coma and death. Its toxicity is due to the high affinity for iron ions (Fe^3+^). As a consequence, intracellular respiration is blocked and lactic acid is excessively secreted. The metabolism of amygdalin in the body is conditioned by many factors, such as digestive enzymes, intestine microbiome, supplementation, and medications [4,5,6,7]. However, amygdalin also has healing properties. The antitumor activity of amygdalin is linked with two hydrolytic enzymes (β-glucosidase and rhodanase). Excessive β-glucosidase and rhodanase deficiency would lead to cell death due to the release and accumulation of toxic hydrogen cyanide. Others, however, mistakenly treated amygdalin as vitamin B_17_, the deficiency of which was supposed to be the cause of cancer. Finally, in vitro studies conducted at the end of the 20th century confirmed the antitumor activity of amygdalin and its beneficial effects on the circulatory, respiratory, and digestive systems. Amygdalin was found to inhibit the proliferation of cancer cells and induce their apoptosis. In addition, it was noted that, at higher concentrations and longer exposure periods, amygdalin was increasingly effective against cervical, prostate, or liver cancer cells. Currently, the use of amygdalin in cancer therapy has its opponents and supporters [8,9,10,11]. The analysis of amygdalin in various matrices (plant material, water, sewage, and biological materials) is being studied by many scientists [12,13,14,15,16]. Xu et al. [12] performed the analysis of amygdalin, neoamygdalin, and amygdalin amide with HPLC-ESI-MS/MS and HPLC-DAD. Juan et al. [17] postulated solid-to-liquid extraction and HPLC/UV determination of amygdalin in apple seeds. Moreover, Feng et al. [18] analyzed amygdalin content in cherry seeds by capillary electrophoresis and Bolarinwa et al. [19] determined its concentration in apple seeds, fresh apples, and apple juices by HPLC. The application of amygdalin as vitamin B_17_ is the focus of a controversial debate. On the one hand, this compound is attributed highly therapeutic effects and is used as an anticancer drug; on the other hand, amygdalin is cataloged as a dangerous substance capable of producing highly toxic effects, due to the release of highly toxic HCN from the molecule in biological processes. Due to the properties of amygdalin, the analysis of this compound is focused on examining its content in two different samples: in food and in materials of animal origin (rat) after consuming products containing amygdalin. Various techniques have been used to determine the amygdalin contents of different foods and biological materials (Table 1).

Li et al. [23] analyzed the pharmacokinetics of amygdalin in rats. Studies of metabolic pharmacokinetics of amygdalin in rats after consumption of feed containing amygdalin are particularly important in order to establish the threshold of pro-health and toxic concentrations. However, a direct analysis of amygdalin from biological materials is difficult due to the interference of other compounds. In addition, there are many ions in this material that form stable complexes with the amygdalin molecule. Therefore, the quantitative determination of amygdalin by electrospray ionization mass spectrometry (ESI-MS) is not possible.

Due to analytical difficulties associated with the analysis of amygdalin, molecularly imprinted polymers (MIPs) were used. MIPs, based on the creation of specific recognition sites in strict polymer networks that have complementary shape, size, and functional groups toward the imprinted molecule, have attracted increasing interest as selective adsorbents for the isolation and enrichment of small organic molecules, metal ions, biomacromolecules, etc. [27,28]. Molecular imprinting of polymers is currently the most generic, versatile, scalable, and cost-effective approach to create synthetic molecular receptors. A number of advantageous properties contribute to the growing interest in MIPs: their high affinity and selectivity, enhanced stability (which is superior to that exhibited by natural biomolecules), and simplicity of their preparation. Recently, special attention has been paid to magnetic MIPs (mag-MIPs). In the new core–shell synthesis of mag-MIPs, a molecularly imprinted polymer surface (shell) covers magnetic iron oxide nanoparticles (core). After the synthesis, the material retains its magnetic properties and thus can be easily manipulated using a neodymium magnet, while providing selectively adsorptive capabilities. Additionally, the decreased size of the mag-MIP particles increases the surface area, enhancing the activity per unit mass of the polymer. Moreover, the recognition sites are located on the surface of the material, facilitating the access of the analyte to the selective cavities and its easy removal. In comparison to the MIPs obtained by traditional synthesis methods, mag-MIPs present a wide set of advantageous properties. Due to the large number of recognition sites located on the surface of MIPs in each mag-MIP particle, the quantification of analytes using these materials is expected to be more sensitive and selective. Mag-MIPs have been successfully used in the analysis of amygdalin by flowing atmospheric-pressure afterglow mass spectrometry (FAPA-MS) [27,28,29,30].

FAPA-MS techniques involve the generation of a direct current or radiofrequency electrical discharge between a pair of electrodes in contact with a flowing inert gas, creating a stream of ionized molecules, radicals, excited state neutral atoms, and electrons. The plasma species are directed toward the sample, resulting in desorption and ionization of the analyte. Ambient plasma MS techniques have many advantages including simple instrumentation, rugged construction of the measuring system, no solvent requirement, and generation of singly charged analyte species that are more easily identifiable than multiple charged ions and various adducts produced by spray-based techniques.

In this paper, we present the results of amygdalin analysis in various matrices (water, sewage, extract, skin, and artificial blood). In addition, we examined the effect of the presence of different ions in the sample on the analysis. We compared the quantitative analysis of amygdalin determination directly and with the use of mag-MIP for pre-concentration of the analyte. The results were obtained by ESI-MS and FAPA-MS.

## 2. Materials and Methods

### 2.1. Chemicals

All reagents used were commercial products. FeCl_2_·4H_2_O, FeCl_3_·6H_2_O, amygdalin, 3-vinyltriethoxysilane (3-VTES), 4-vinylpyridine (4-VP), ethylene glycol dimethacrylate (EGDMA), 2,2-azobisisobutyronitrile (AIBN), ammonia solution, acetonitrile, dimethyl sulfoxide (DMSO), acetone, and methanol were purchased from Merck (Darmstadt, Germany). Formic acid, acetic acid, hydrochloric acid, sodium chloride, potassium chloride, sodium hydroxide, calcium perchlorate, lead perchlorate, copper (II) nitrate (V), and cadmium nitrate (V) tetrahydrate were obtained from POCH (Gliwice, Poland). Buffer solutions—pH 2 (hydrochloric acid, sodium chloride, and aminoacetic acid), pH 7 (disodium hydrogen phosphate, and citric acid), and pH 10 (boric acid, potassium chloride, and sodium hydroxide)—were from Eurochem BGD (Tarnów, Poland). Transparent tape 3M (19 mm × 65.8 m) from Scotch (Minneapolis, MN, USA) was used for skin testing. Certified organic sweet apricot kernels containing 30 mg of amygdalin per 4.8 g material (6.25 mg g^−1^) were from Sunfood (El Cajon, CA, USA). Bitter almonds were from Uzbekistan, purchased from Skworcu (Siemianowice Śląskie, Poland). The apples and apricots came from the local market (Poznań, Poland). Human serum from male AB plasma USA origin, sterile-filtered were purchased from Merck (Darmstadt, Germany) was used as the biological material.

### 2.2. Synthesis

#### 2.2.1. Fe_3_O_4_

Magnetite nanoparticles (Fe_3_O_4_) were synthesized by using FeCl_2_ 4H_2_O and FeCl_3_ 6H_2_O as precursors. FeCl_2_ 4H_2_O (2.0 g) and FeCl_3_ 6H_2_O (5.2 g) were dissolved in 25 mL deoxygenated water, and then 0.85 mL of concentrated HCl was added. The resulting solution was added dropwise into 250 mL of 1.5 M NaOH solution upon vigorous stirring and N_2_ protection at 80 °C. The synthesized magnetic nanoparticles (MNPs) were separated from the solution by a powerful magnet and washed with 200 mL deionized water three times. Fe_3_O_4_ was used for the preparation of a stable aqueous suspension. A total of 10 mL of an aqueous solution of citric acid (0.5 g mL^−1^) was added to the vigorously stirred suspension of washed nanoparticles. The pH value was set to 5.2 with a concentrated ammonia solution and heated to 80 °C. After 90 min, the pH value of the solution was elevated to 10. Lastly, the suspension was centrifuged for 5 min at 4000 rpm to remove any agglomerated nanoparticles. The obtained MNPs were dried at 60 °C.

#### 2.2.2. Fe_3_O_4_@SiO_2_@VIN

Fe_3_O_4_ (2.4 g) and 125 mL DMSO were added to a round bottom flask and placed in an ultrasonic bath for 1 h. 3-VTES (12 mmol) was slowly added to the suspension. The mixture was stirred using a magnetic stirrer for 24 h. Then, the precipitate was isolated with a magnet and washed with distilled water and acetone. The resulting product Fe_3_O_4_@SiO_2_@VIN (Fe_3_O_4_ as a magnetic core covered with a thin layer of silica and vinyl (VIN) groups) was dried.

#### 2.2.3. Fe_3_O_4_@SiO_2_@VIN@MIP

Amygdalin (1 mmol) as a template molecule and 4-VP (4 mmol) as a functional monomer were dissolved in 50 mL acetonitrile and 35 mL methanol. Subsequently, 20 mmol of EGDMA (previously purified with aluminum oxide, activated, basic, Brockman I) as a cross-linking agent and 1 g of Fe_3_O_4_@SiO_2_@VIN were added. The ratio of the template:monomer:cross-linking agent was 1:4:20. The pre-polymerization solution was sonicated and purged with nitrogen for 30 min. Afterward, 1 mL AIBN as initiator was added. The tube was sonicated and purged with nitrogen for an additional 5 h at 70 °C, then sealed and placed in an oven for 24 h at 50 °C. After polymerization, Fe_3_O_4_@SiO_2_@VIN@MIP-amygdalin was dried under reduced pressure and grounded. Amygdalin was extracted with acetonitrile via Soxhlet extraction for 24 h. Then, the final product Fe_3_O_4_@SiO_2_@VIN@MIP was dried at 60 °C under vacuum and grounded.

### 2.3. Instruments

**Figure 2:**
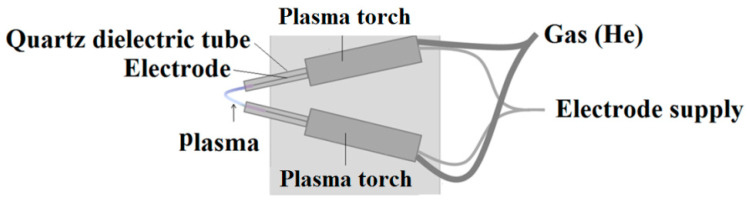
Scheme of V-FAPA (flowing atmospheric-pressure afterglow) ambient plasma source.

ESI-MS and ESI-MS^n^ spectra were recorded using an amaZon SL ion trap (Bruker, Bremen, Germany) equipped with an electrospray ion source in infusion mode. The sample solution was introduced into the ionization source at a flow rate of 5 μL min^−1^ using a syringe pump. The apparatus was operated using the so-called “enhanced resolution mode” (mass range: 50–2200 *m*/*z*, scanning rate: 8100 *m*/*z* per second). The capillary voltage was set at −4.5 kV and the end plate offset was at −500 V. The source temperature was 80 °C and the desolvation temperature was 250 °C. Helium was used as the cone gas and desolvating gas (nitrogen) at flow rates of 50 L h^−1^ and 800 L h^−1^, respectively. The mass spectrometer was operated in the ESI positive and negative ionization mode. In MS^n^ experiments, the width of the selection window was set at 2 Da and the amplification of the excitation was set according to the experiment (from 0.2 to 1.5 V). Mass spectrometers were equipped optionally in a V-FAPA (Figure 2) NOVA011 ambient plasma source (ERTEC, Wroclaw, Poland).

**Figure 3:**
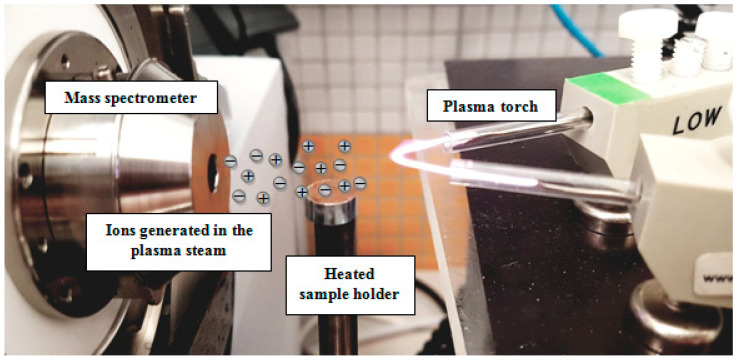
The measuring system: plasma torch, heated sample holder, and amaZon SL mass spectrometer.

V-FAPA was used to generate plasma, allowing the ionization of analyte particles released thermally from a heated crucible with temperature regulation from 20 to 400 °C, at a temperature increase rate of 3 °C s^−1^. The temperature of maximum desorption of the analyte was 340 ± 10 °C. The mini crucible allowing the temperature-controlled desorption was placed approx. 10 mm below the ion stream. The distance between the inlet to the mass spectrometer and the FAPA ion source was about 10 cm (Figure 3). The experimental details of the FAPA method were presented in previous publications [29,31].

The amygdalin content of the solution was determined using a chromatography system (Alliance, type 2690, Waters, Milford, MA, USA) coupled to a UV photodiode array and MS detectors. Quantification was performed using a C_18_ column (Atlantis T3 column, silica-based, reversed-phase, 100 Å, 3 µm, 3 mm × 100 mm, 1/pk from Waters (Warsaw, Poland)) at 25 °C. The wavelength range in the UV spectrum was 200–500 nm and the range of recorded masses was 100–1000 *m*/*z*. Chromatography was performed using a flow rate of 0.5 mL per minute and an isocratic eluent program, and a mixture of 25% volume of methanol and 75% volume of water was used as the eluent. The injector volume was 10 µL and the total elution time was 20 min.

The X-ray fluorescence (XRF) spectra were produced on a PANalytical MiniPal2 XRF spectrometer (Malvern, UK) equipped with a rhodium X-ray vacuum tube under the given conditions: time of the analysis, 200 s and X-ray tube voltage, 15 kV. The current generated in the electron beam varied between 20 and 53 µA.

X-ray photoelectron spectroscopy (XPS) was utilized to evaluate the chemical composition of the investigated MIPs. The measurements were carried out on an Escalab 250Xi spectroscope (Thermo Fisher Scientific, Waltham, MA, USA). The XR 50 source was used, and 60 eV pass energy and 90 eV bias voltage were utilized. The peak deconvolution was carried out using the Avantage software provided by the spectroscope manufacturer.

All measurements were made three times. The presented results are average values.

### 2.4. Analysis

#### 2.4.1. Analysis of Amygdalin in Aqueous Solutions by ESI-MS

The amygdalin solutions were prepared at concentrations ranging from 0.045 μg L^−1^ to 45 mg L^−1^. ESI-MS measurements were performed in buffer solutions with pH 2, 7, and 10. The linear range of the method, limit of detection (LOD), and limit of quantification (LOQ) were determined.

To study the effect of ions present in the solution on the determination of amygdalin by ESI-MS, the following ions were added separately to 10 mL of 4.5 mg L^−1^ amygdalin solutions: Cu^2+^, Pb^2+^, Ca^2+^, Na^+^, and K^+^ in a molar ratio of 1:1 relative to amygdalin.

#### 2.4.2. Analysis of Amygdalin in Aqueous Solutions by FAPA-MS

The amygdalin solutions were prepared at concentrations ranging from 0.045 μg L^−1^ to 45 mg L^−1^. FAPA-MS measurements were performed in a buffer solution with pH 7. The linear range of the method, LOD, and LOQ were determined.

#### 2.4.3. Binding and Release of Amygdalin from Fe_3_O_4_@SiO_2_@VIN@MIP/Fe_3_O_4_@SiO_2_@VIN@MIP-Amygdalin in Aqueous Solutions

In order to investigate the binding of amygdalin by Fe_3_O_4_@SiO_2_@VIN@MIP in aqueous solutions, three amygdalin concentrations of 0.0045 mg L^−1^, 0.45 mg L^−1^, and 45 mg L^−1^ were prepared. Then, 20 mg of Fe_3_O_4_@SiO_2_@VIN@MIP was added to 5 mL of each solution and stirred for 1 h. Subsequently, Fe_3_O_4_@SiO_2_@VIN@MIP-amygdalin was removed from the solution. To determine the amount of non-adsorbed amygdalin by Fe_3_O_4_@SiO_2_@VIN@MIP, the remaining amygdalin in solutions was measured by ESI-MS. The amount of adsorbed amygdalin in Fe_3_O_4_@SiO_2_@VIN@MIP-amygdalin was measured by FAPA-MS.

To test the degree of amygdalin release from Fe_3_O_4_@SiO_2_@VIN@MIP-amygdalin, 100 mg of empty Fe_3_O_4_@SiO_2_@VIN@MIP was placed in aqueous solutions containing 2 mg, 5 mg, and 7 mg of amygdalin. After all the amygdalin was bound to the polymer structure, Fe_3_O_4_@SiO_2_@VIN@MIP-amygdalin was isolated from the solution, washed, and dried. The three Fe_3_O_4_@SiO_2_@VIN@MIP-amygdalin polymers prepared in this way containing respectively 2, 5, and 7 mg of amygdalin per 100 mg of polymer were placed in 10 mL of an aqueous solution, acidified with formic acid to pH 2, and intensively stirred for 1 h to completely release amygdalin. After this time, both the solution (ESI-MS method) and Fe_3_O_4_@SiO_2_@VIN@MIP (FAPA-MS method) were analyzed for amygdalin content.

#### 2.4.4. Extraction of Amygdalin from Fruit Seeds

##### Water Extraction at 40 °C

Bitter almond kernels were grounded in a blender and 2 g was weighed into a conical flask (200 mL). Water (50 mL) was added and the flask was placed in a shaker with temperature control. Amygdalin extractions were carried out for 120 min. The extracts were filtered and analyzed with two methods. In the first method, the analysis of the amygdalin water extract with the ESI-MS method was performed. In the second method, 10 mg of Fe_3_O_4_@SiO_2_@VIN@MIP was added to the solution to pre-concentrate the analyte (amygdalin), followed by a direct analysis of amygdalin from the polymer structure using FAPA-MS. The amygdalin determination process was repeated three times. Apple and apricot extracts were prepared and analyzed identically.

##### Ethanol Extraction at 40 °C

The ethanol extraction was performed identically as described above, with ethanol used as a solvent, instead of water.

#### 2.4.5. Analysis of Amygdalin Skin Penetration

In order to analyze amygdalin found on the skin, hands were accidentally stained with amygdalin solution during work, and the tape-stripping technique was used [32,33,34]. The 3M adhesive tape was used to collect the skin sample. The tape (1 cm^2^) was glued to the palm and after 1 min it was torn off. Then, the tape containing the test substance was immersed in ethanol (5 mL) and left for 1 h. After the set time had elapsed, 200 μL of solution for ESI-MS analysis was taken. The tape was glued four more times in the same place. The protocol was approved by the Ethics Committee (Komisja Bioetyczna przy Uniwersytecie Medycznym im. Karola Marcinkowskiego w Poznaniu, 469/16, 16 June 2016) and informed consent was obtained. All measurements were taken three times.

#### 2.4.6. Analysis of Amygdalin in Human Serum

To determine amygdalin in human serum from human male AB plasma, 5 mg of amygdalin was added to 1 mL human serum. The ESI-MS spectrum was recorded.

## 3. Results and Discussion

### 3.1. Analysis of Amygdalin in Aqueous Solutions by ESI-MS

The analysis of amygdalin in aqueous solutions by ESI-MS showed signals with different *m*/*z* values, depending on the pH value of the solution.

**Figure 4:**
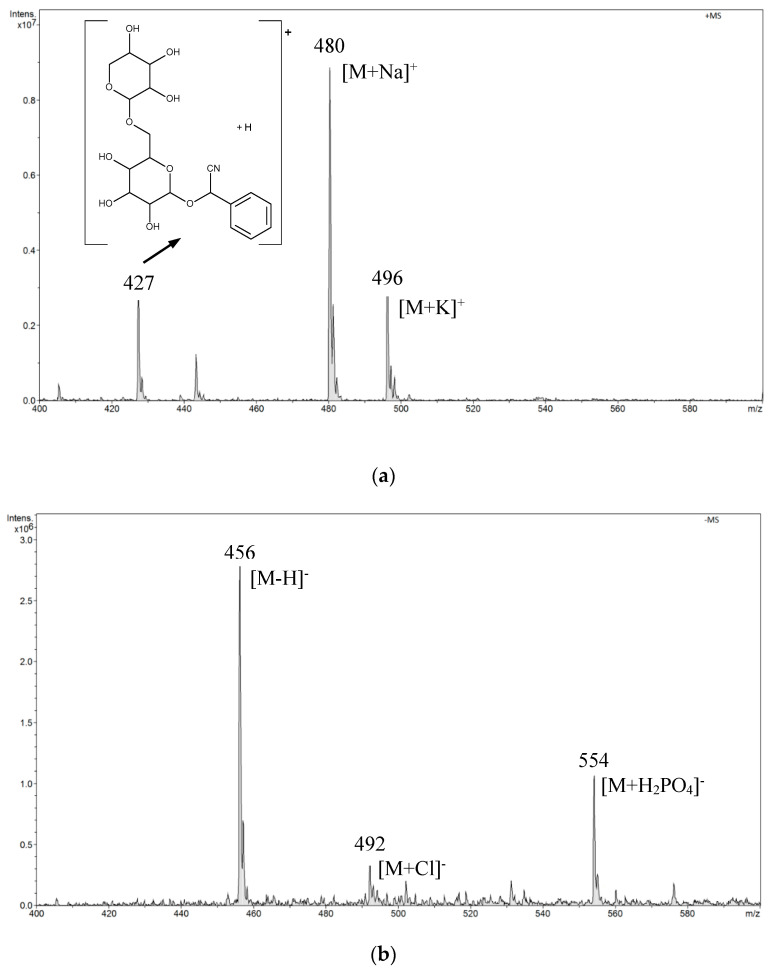
The electrospray ionization mass spectrometry (ESI-MS) spectrum of amygdalin performed in a buffer solution of pH 7.0 (disodium hydrogen phosphate and citric acid): (**a**) positive mode, (**b**) negative mode.

In the positive mode ESI-MS spectrum for amygdalin in a buffer solution of pH 7.0, we observed *m*/*z* 480 [M + Na]^+^ and *m*/*z* 496 [M + K]^+^ signals and the *m*/*z* 427 signal from the fragmentation ion. The fragment ion structure is shown in the spectrum in Figure 4a. In the negative mode ESI-MS spectrum, we observed signals *m*/*z* 456 [M − H]^−^, *m*/*z* 492 [M + Cl]^−^, and *m*/*z* 554 [M + H_2_PO_4_]^−^. This was a consequence of measurements in the buffer in which the component was disodium hydrogen phosphate (Figure 4b).

**Figure 5:**
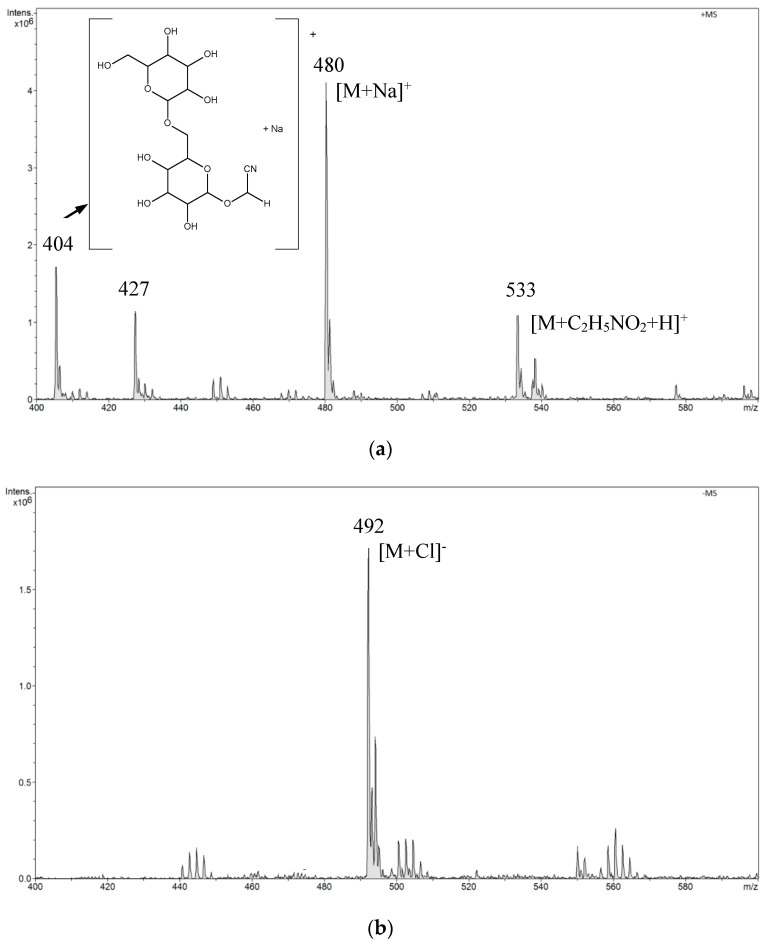
The ESI-MS spectrum of amygdalin performed in a buffer solution of pH 2.0 (hydrochloric acid, sodium chloride, and aminoacetic acid): (**a**) positive mode, (**b**) negative mode.

In the positive mode ESI-MS spectrum for amygdalin in a buffer solution of pH 2.0, we observed the main signal *m*/*z* 480 [M + Na]^+^ and a signal of lower intensity *m*/*z* 533. The *m*/*z* 533 signal resulted from the attachment of aminoacetic acid present in the buffer to the amygdalin molecule. We also observed signals *m*/*z* 427 and *m*/*z* 404 from the fragmentation ions (Figure 5a). The structure of the fragmentation ion *m*/*z* 404 is presented in the spectrum in Figure 5a. In the negative mode ESI-MS spectrum, we observed only one signal *m*/*z* 492 [M + Cl]^−^ (Figure 5b).

**Figure 6:**
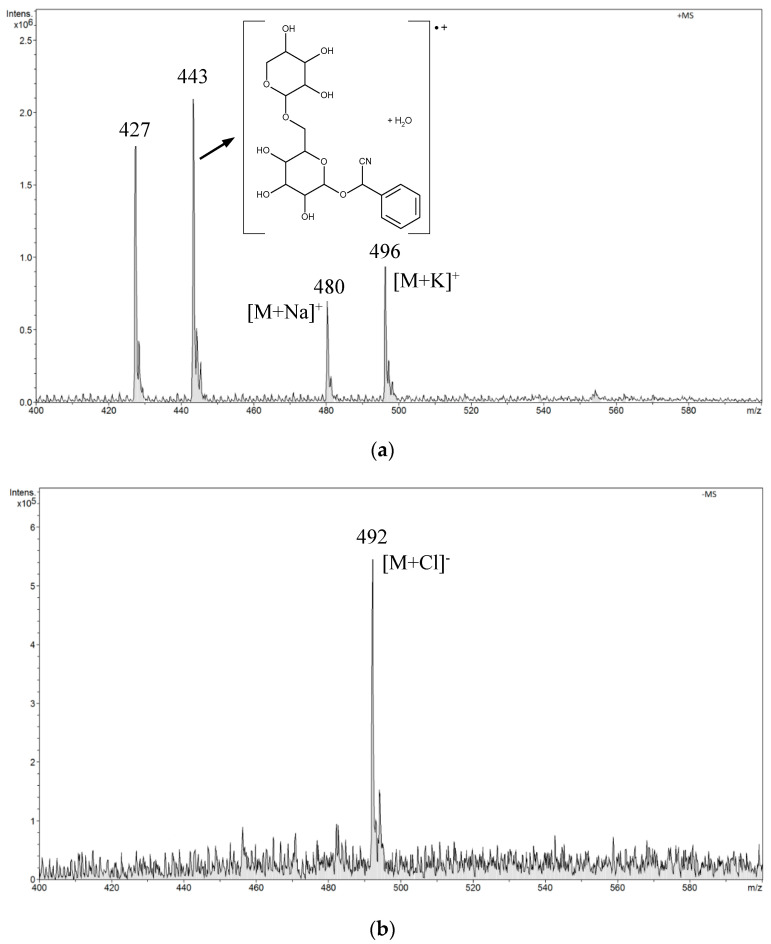
The ESI-MS spectrum of amygdalin performed in a buffer solution of pH 10.0 (boric acid, potassium chloride, and sodium hydroxide): (**a**) positive mode, (**b**) negative mode.

In the positive mode ESI-MS spectrum for amygdalin in a buffer solution of pH 10.0, we observed two low-intensity signals *m*/*z* 480 [M + Na]^+^ and *m*/*z* 496 [M + K]^+^ and two signals from the fragmentation ions (*m*/*z* 427 and *m*/*z* 443). The structure of the fragmentation ion *m*/*z* 443 is shown in the spectrum in Figure 6a. In the negative mode ESI-MS spectrum, we observed only one signal *m*/*z* 492 [M + Cl]^−^ (Figure 6b).

**Figure 7:**
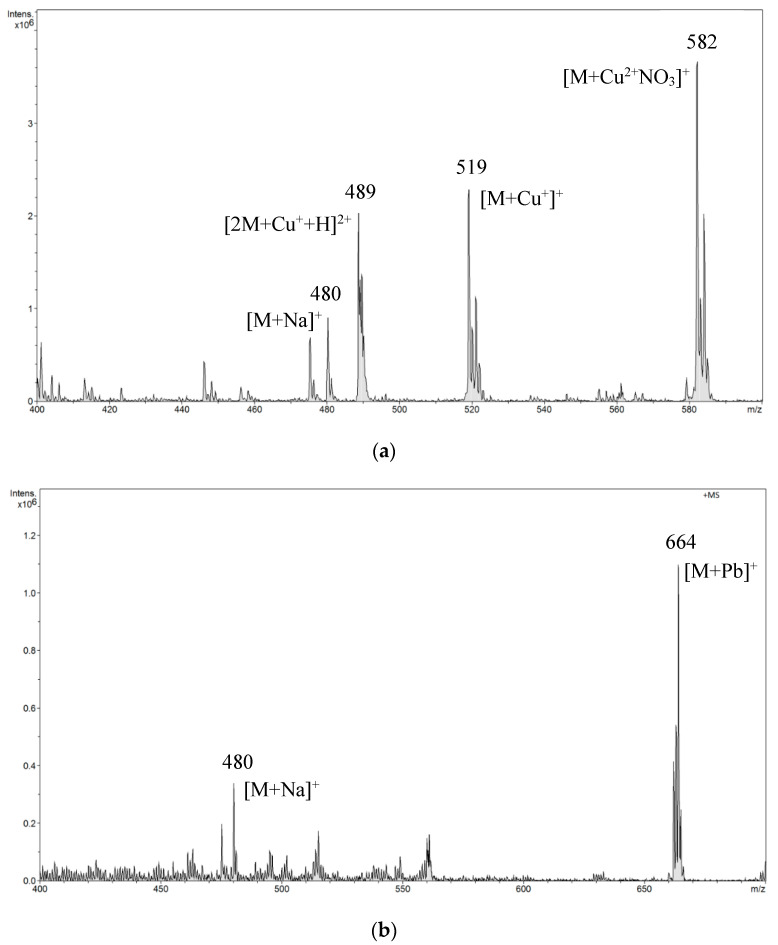

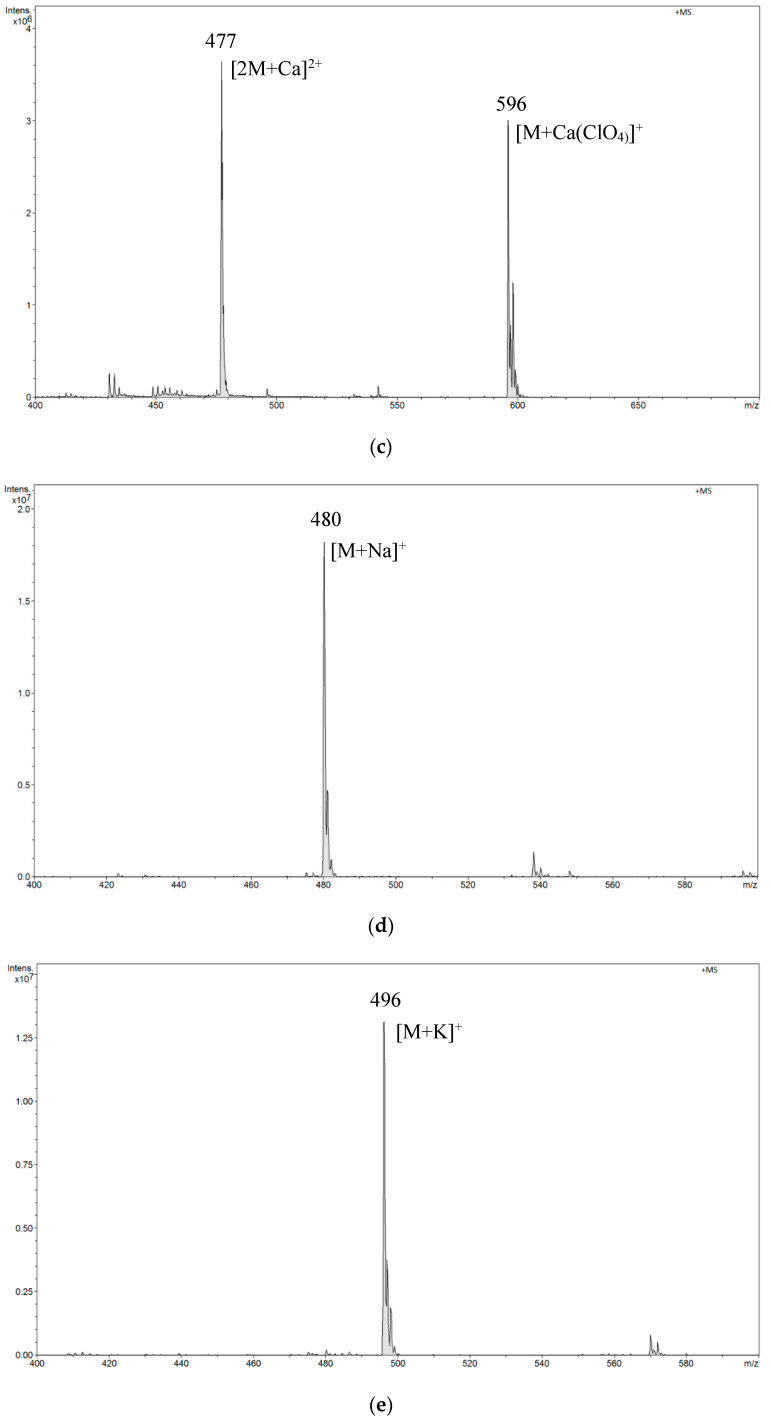
The ESI-MS spectrum of amygdalin performed in solutions with the addition of various ions: (**a**) Cu^2+^, (**b**) Pb^2+^, (**c**) Ca^2+^, (**d**) Na^+^, (**e**) K^+^.

Based on the obtained results, further tests were carried out in neutral solutions. The presence of other components in the sample composition also affects the determination of amygdalin in environmental samples. Amygdalin easily complexes ions found in solution, which we took into account in the conducted research. Solutions with the addition of various ions (Cu^2+^, Pb^2+^, Ca^2+^, Na^+^, and K^+^) were analyzed (Figure 7a–e). The results are shown in the spectra. In the sample with both amygdalin and copper ions, a plurality of complexes were formed. The ions formed as a result of attaching ubiquitous sodium in the environment were still visible. They were also present in the sample with added lead ions. Furthermore, amygdalin with lead ions also formed complexes. In the remaining samples, amygdalin also formed complexes with calcium, sodium, or potassium ions. The tested ions are very common in the environment. They occur naturally in various biological materials. The presence of these ions in the analyzed sample practically prevented the correct analysis of amygdalin using the ESI-MS method. The ESI-MS spectra reflected the constituents present in solution; however, this process may be limited for several reasons. The changing solution conditions during droplet evaporation in a spectrometer, such as changes in pH, ionic strength, unequal evaporation of constituents, or occurrence of charge reduction, may alter the metal complex or the equilibrium conditions. Moreover, species which are stable in solution may not be stable in the gas phase due to the absence of a solvent [35,36,37]. In the case of metal–amygdalin complexes where the structure is determined by electrostatic interactions, the ESI-MS spectra reflect the composition of the solution [26]. Electrostatic interactions are greatly strengthened in a solvent environment. As a consequence, metal–ligand complexes with strong electrostatic interactions are more stable in the gas phase [38]. In addition, as a consequence of the dynamic character of liquid-based ionization in the ESI process, it is possible in this process to monitor liquid-phase reactions of metal ions in solution [39].

A linear relationship between signal intensity and amygdalin concentration was established by selecting *m*/*z* 458 [M + H]^+^, *m*/*z* 480 [M + Na]^+^, and *m*/*z* 496 [M + K]^+^ as analytical signals. The linearity range of the method was 4.5 µg L^−1^–45 mg L^−1^ in positive mode ESI-MS. The LOD for ESI-MS was 0.101 ± 0.003 µg L^−1^ and the LOQ was 0.303 ± 0.009 µg L^−1^.

### 3.2. Analysis of Amygdalin in Aqueous Solutions by FAPA-MS

**Figure 8:**
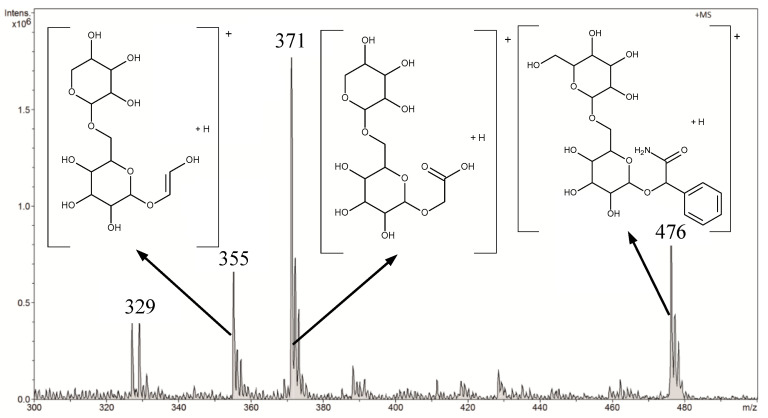
The positive mode FAPA-MS spectrum of amygdalin in 45 mg L^-1^ aqueous solution.

Amygdalin was determined in aqueous solutions in the range from 0.045 μg L^−1^ to 45 mg L^−1^. In the FAPA-MS method, tests were performed in negative and positive ion modes. After a preliminary analysis of the results, the rest of the measurements were performed in the FAPA-MS positive ion mode. In FAPA-MS mass spectra, *m*/*z* 371 and *m*/*z* 476 signals were observed. These signals were derived from the reaction products of amygdalin with water in the plasma stream and the products of hydrolysis and fragmentation. As a result of the hydrolysis of nitriles, amides were formed (Equation (1)). Amides can be hydrolyzed to carboxylic acids. Moreover, the molecule can fragment (Equation (2)). Structural formulas of compounds are presented in the mass spectrum in Figure 8.

(1) $$R_{1}-\mathrm{CN}\;\overset{\mathrm{hydrolisis}}{\to }\;R_{1}-{\mathrm{CONH}}_{2}$$

(2) $$R_{1}-\mathrm{CONH}\;\overset{\mathrm{hydrolisis}}{\to }\;R_{1}-\mathrm{COOH}\;\overset{\mathrm{fragmentation}}{\to }\;R_{2}-\mathrm{COOH}$$

The *m*/*z* 371 signal was selected as the analytical signal. The linearity range was 4.5 µg L^−1^–45 mg L^−1^ for FAPA-MS. The LOD was 0.050 ± 0.002 µg L^−1^ and the LOQ was 0.150 ± 0.006 µg L^−1^.

### 3.3. Comparison of Analytical Parameters of the ESI-MS and FAPA-MS Methods

To compare the methods of determining amygdalin in aqueous solutions, ESI-MS and FAPA-MS techniques were analyzed and the results obtained are presented in Table 2.

**Figure 9:**
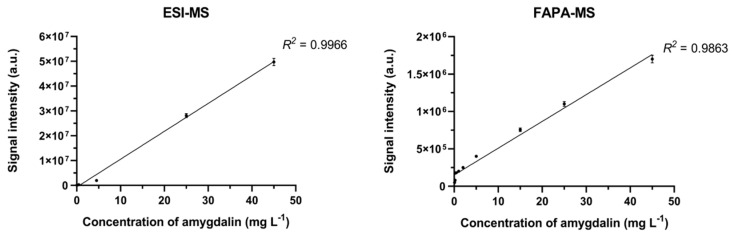
Dependence of signal intensity vs. amygdalin concentration in the sample.

Both the methods were characterized by similar values of analytical parameters (Figure 9): the scatter of results and the average value of the measurement in relation to the tested systems (Table 3).

The FAPA-MS method had lower LOD, but the linearity range for FAPA-MS and ESI-MS was the same. The attractiveness of the FAPA-MS method in relation to ESI-MS lies in the possibility of analyzing amygdalin directly from Fe_3_O_4_@SiO_2_@VIN@MIP-amygdalin. This eliminates the influence of interferants, concentrates the sample, and facilitates the analysis of real samples with a low concentration of the analyte in the presence of other compounds in the sample.

### 3.4. Comparison of the HPLC-UV/MS and FAPA-MS Methods with Certified Reference Materials of Apricot Kernels Containing Amygdalin

**Figure 10:**
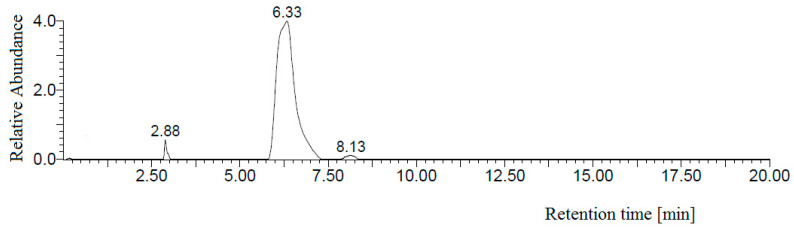
HPLC-diode array chromatogram of amygdalin solution (0.5 mg mL^−1^).

**Figure 11:**
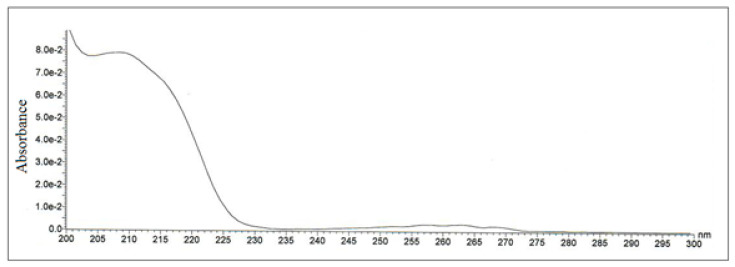
UV spectrum of amygdalin, HPLC retention time t_R_ = 6.33 min.

**Figure 12:**
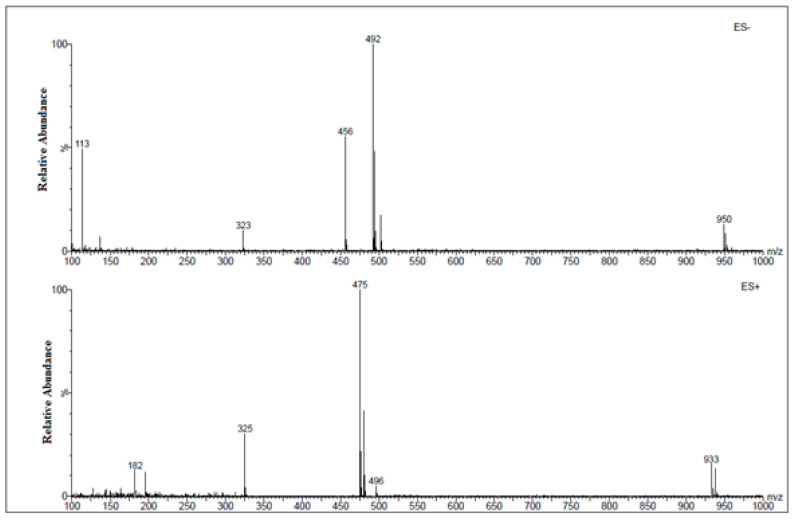
Full scan spectrum of amygdalin, HPLC retention time t_R_ = 6.33 min, *m*/*z* 456 [M − H]^−^, *m*/*z* 950 [2M + Cl]^−^, *m*/*z* 492 [M + Cl]^−^, *m*/*z* 475 [M + Na]^+^, *m*/*z* 496 [M + K]^+^, and *m*/*z* 938 [2M + Na]^+^.

**Figure 13:**
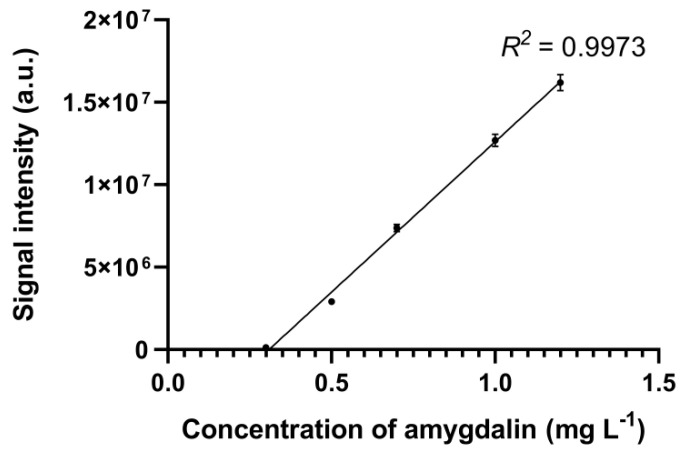
Dependence of signal intensity vs. amygdalin concentration in the sample.

For the quantification of amygdalin content in solution, the calibration curve for HPLC-UV/MS was constructed using five different solutions of amygdalin in methanol (Figure 10, Figure 11, Figure 12 and Figure 13). The chromatogram of amygdalin solution (0.5 mg mL^−1^) is presented in Figure 10.

A good regression equation (*R*^2^ = 0.9973) for HPLC-UV/MS determination of amygdalin was obtained, where the signal intensity of amygdalin was denoted in the *y*-axis and amygdalin concentration (mg mL^−1^) was denoted in the *x*-axis (Figure 13).

In order to validate the method of determination of amygdalin in plant materials, Sunfood’s certified organic sweet apricot kernels containing 30 mg of amygdalin per 4.8 g material (6.25 mg g^−1^) were used. The apricot kernels (4.8 g) were extracted with 50 mL of water or ethanol, and amygdalin concentration in the extracts was determined by HPLC-UV/MS and FAPA-MS using the Fe_3_O_4_@SiO_2_@VIN@MIP material for the selective concentration of the analyte.

A comparison of the results of HPLC-UV/MS and FAPA-MS methods for the determination of amygdalin in apricot kernels is presented in Table 4.

The applied methods showed that, in the case of extraction of amygdalin with water or ethanol, followed by determination with HPLC-UV/MS, the results were lower than those declared in the certified material. On the other hand, in the case of extraction with water or ethanol, followed by selective adsorption by Fe_3_O_4_@SiO_2_@VIN@MIP and FAPA-MS determination, the results were higher than those declared in the certified material.

### 3.5. Characteristic of Amygdalin Binding and Release from Fe_3_O_4_@SiO_2_@VIN@MIP/Fe_3_O_4_@SiO_2_@VIN@MIP-Amygdalin in Aqueous Solutions

**Figure 14:**
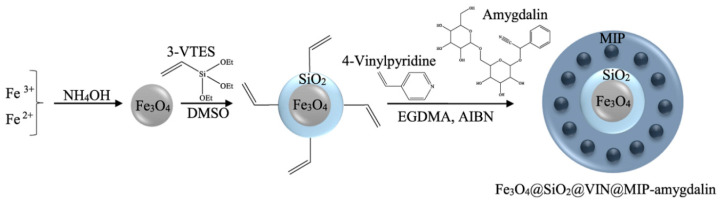
Scheme of Fe_3_O_4_@SiO_2_@VIN@MIP synthesis.

As a result of multistage synthesis, the hybrid material Fe_3_O_4_@SiO_2_@VIN@MIP was obtained (Figure 14).

**Figure 15:**
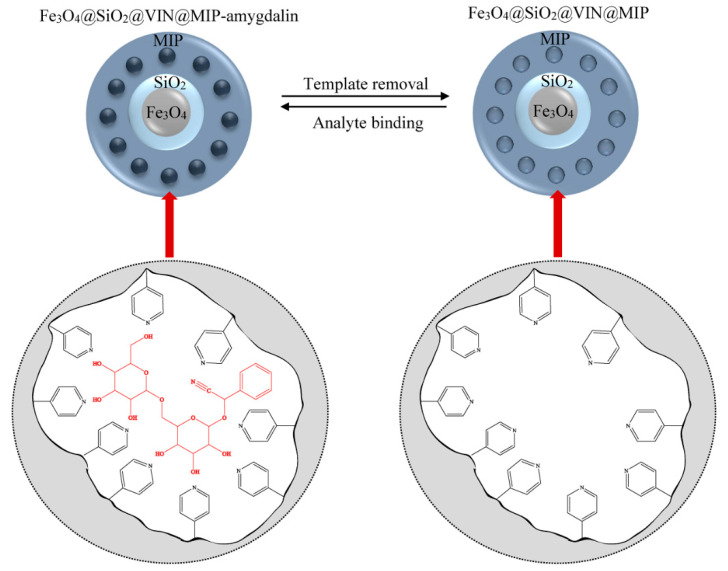
Release and binding of amygdalin in Fe_3_O_4_@SiO_2_@VIN@MIP-amygdalin/Fe_3_O_4_@SiO_2_@VIN@MIP.

After removing the template, the resulting system was capable of binding analyte–amygdalin according to the equilibrium shown in Figure 15.

**Figure 16:**
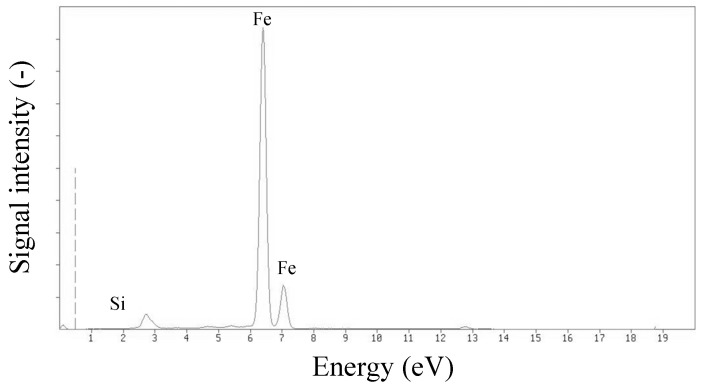
X-ray fluorescence (XRF) spectrum of Fe_3_O_4_@SiO_2_@VIN@MIP.

XRF analysis confirmed that a significant part of the Fe_3_O_4_@SiO_2_@VIN@MIP mass consisted of a magnetic core (Fe_3_O_4_) covered with a thin silica coating. The mass ratio of Fe:Si was approximately 100:1 (Figure 16).

Moreover, we performed XPS analyses for Fe_3_O_4_@SiO_2_@VIN@MIP and Fe_3_O_4_@SiO_2_@VIN@MIP-amygdalin to confirm the structure of the formed core–shell particles. The results of XPS analyses are collectively presented in Figures S1 and S2. The XPS analyses were surface tests and the surface of the obtained materials was a polymer coating. The low-intensity signal from the magnetic core formed by Fe_3_O_4_ confirmed that it was covered with a thin layer of silica and then a polymer layer. The observed signals came mainly from carbon and oxygen, i.e., elements building the polymer structure. Furthermore, we observed a slight difference in oxygen and nitrogen content between empty Fe_3_O_4_@SiO_2_@VIN@MIP and the polymer with amygdalin. It was an additional confirmation that during the synthesis, the polymer bound amygdalin molecules in its structure, and during extraction, the molecules were successfully washed away.

**Figure 17:**
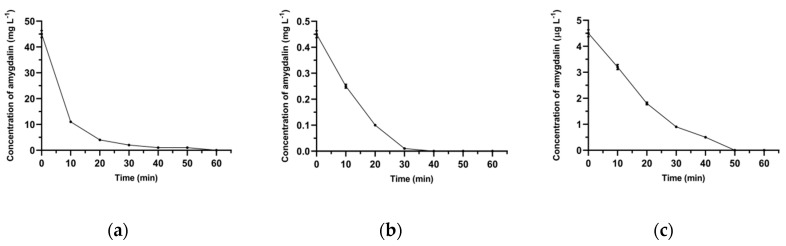
The concentration of amygdalin over time in solution after addition of Fe_3_O_4_@SiO_2_@VIN@MIP. C_0_ = 45 mg L^−1^ (**a**), 0.45 mg L^−1^ (**b**), and 4.5 μg L^−1^ (**c**).

In the first method, ESI-MS was used to examine the decrease in amygdalin concentration in aqueous solutions after the addition of Fe_3_O_4_@SiO_2_@VIN@MIP. The binding of amygdalin by the polymeric material was performed for three different initial concentrations of amygdalin solutions. The decrease in amygdalin concentration in these solutions over time is shown in Figure 17.

In solutions with different concentrations of amygdalin, it was most effectively bound in the polymer structure at different time points. However, for all samples, the total binding time of the analyte did not exceed 1 h; therefore, 1 h was considered as the optimal binding time of the analyte from the real samples. All experiment times with the use of mag-MIP were assumed to be 1 h. The second FAPA-MS method examined the content of amygdalin in Fe_3_O_4_@SiO_2_@VIN@MIP-amygdalin isolated from complementary aqueous solutions of amygdalin. The parallel determination of the analyte by the FAPA-MS method was carried out directly from the polymer structure. Due to the magnetic properties of the material, it was easily isolated from solutions using a small neodymium magnet. Then, the polymer was washed three times with water. In the next step, a magnet was placed in the FAPA-MS measuring system and a direct analysis of amygdalin from the polymer structure was performed. The complementary results obtained with both methods are presented in Table 5. The Fe_3_O_4_@SiO_2_@VIN@MIP polymer bound amygdalin from the solution very efficiently, and the amygdalin content determined by the FAPA-MS method directly from the polymer structure corresponded to the amount of the analyte in the test sample.

The resulting Fe_3_O_4_@SiO_2_@VIN@MIP showed high amygdalin-binding capacity in aqueous solutions of 11.5 mg amygdalin/g material.

**Figure 18:**
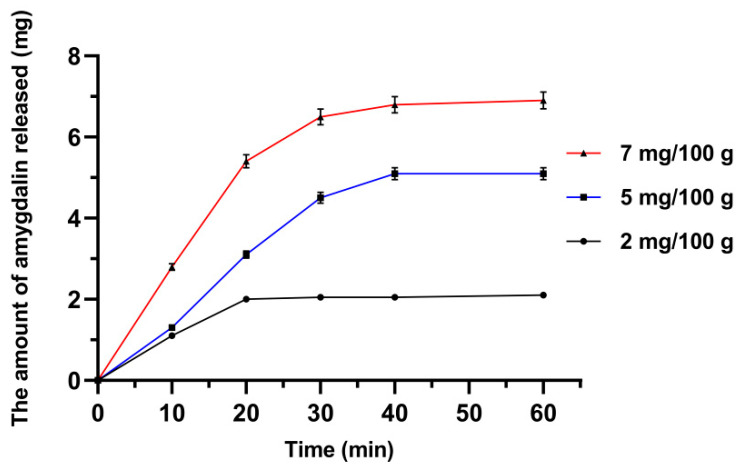
The amount of amygdalin released over time in solution after addition Fe_3_O_4_@SiO_2_@VIN@MIP-amygdalin.

The process of amygdalin release from Fe_3_O_4_@SiO_2_@VIN@MIP-amygdalin in an aqueous solution was also analyzed. The release of the analyte into the solution was performed from three polymer matrices containing different amounts of amygdalin. The analysis was carried out for 1 h, after which time the amygdalin content in the solution did not increase. The optimal time for the complete release of amygdalin from the material was 1 h. In the first method, the increase in amygdalin concentration in the solution was examined by ESI-MS. The results are shown in Figure 18.

After 1 h, Fe_3_O_4_@SiO_2_@VIN@MIP was isolated from the solution, washed with water, and the remaining amygdalin with the polymer material was analyzed by FAPA-MS. The results are shown in Table 6.

The amygdalin bound in the polymer structure in the solution was released practically completely within 1 h. The amount of unreleased amygdalin did not exceed 1% of the initial value; thus, it did not significantly affect the obtained results.

### 3.6. The Use of Hybrid Material Fe_3_O_4_@SiO_2_@VIN@MIP in the Analysis of Amygdalin in Samples

In order to check the usefulness of the proposed analytical method, the content of amygdalin in aqueous and ethanolic extracts of bitter almonds by ESI-MS was determined. The material Fe_3_O_4_@SiO_2_@VIN@MIP was used as a molecular scavenger to pre-concentrate amygdalin from aqueous solutions. In the next step, the obtained magnetic scavenger was used to pre-concentrate amygdalin in the water and alcohol extracts, and a direct analysis on the polymer material by the FAPA-MS method was performed. The obtained results are presented in Table 7.

The concentration of amygdalin in bitter almonds was 22.1 mg/g in the aqueous extract and 37.9 mg/g in the ethanol extract, as determined by ESI-MS. When using the FAPA-MS method in combination with Fe_3_O_4_@SiO_2_@VIN@MIP, the content was about 26% higher. The analysis of amygdalin in bitter almonds allowed to design an analytical method. Then, in the same way, extracts of apple and apricot seeds were prepared. Using the FAPA-MS method combined with Fe_3_O_4_@SiO_2_@VIN@MIP, it was determined that the amygdalin content in apple seeds and apricot seeds ranged from 0.87 to 1.43 mg/g and from 1.59 to 2.98 mg/g, respectively. The obtained results were compared with the results obtained using the HPLC-UV method by Bolarinwa et. al. [1], which showed that the presented method gave complementary results to the results obtained by other researchers.

### 3.7. Analysis of Amygdalin Skin Penetration

Using the tape-stripping technique, the amount of amygdalin that penetrates the skin when poured in an aqueous solution was determined (Table 8).

Based on the measurements, it was found that amygdalin from aqueous solutions very poorly penetrated subsequent layers of the skin. As much as 99.7% of amygdalin was found in the epidermis layer.

### 3.8. Analysis of Amygdalin in Human Serum

**Figure 19:**
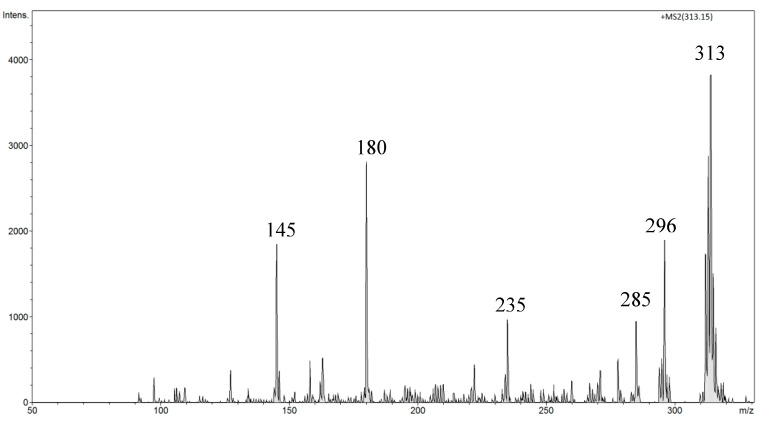
FAPA-MS^2^ fragmentation spectrum of the *m*/*z* 313 ion observed for amygdalin.

Studies show that amygdalin in mammals undergoes enzymatic hydrolysis and is converted to two glucose molecules (glucose and prunasin) as well as mandelonitrile, which due to its unstable nature, is spontaneously converted to HCN and benzaldehyde [40,41,42]. We used artificial blood human serum, from human male AB plasma, sterile-filtered and fortified with amygdalin to establish the level of detection of this analyte in this biological matrix. This human AB serum is used in tissue engineering, transplantation, and cell therapy applications. The fortification was performed at the level of 5 mg of amygdalin per 1 mL of human serum. The toxicity of amygdalin through oral administration is higher than intravenous route. After oral administration, amygdalin produces more toxic hydrocyanic acid in the organism. In rats, the fatal dose (LD_50_) of oral administration of amygdalin is 880 mg kg^−1^ body weight [43]. The human lethal dose for intravenous injection is 5 g. Humans can present systemic toxicity after oral administration of amygdalin, 4 g per day for half a month or intravenous injection for a month. Moreover, the digestive system toxicity response is more common, with changes in atrial premature beats and electrocardiogram T wave. The toxicity response above can disappear after drug withdrawal. If the dose is reduced to daily oral doses of 0.6–1 g, toxicity can be avoided [44]. In the FAPA-MS spectra of human serum fortified with amygdalin, the signals *m*/*z* 371 (at 200 °C) and *m*/*z* 313 (at 340 °C) were observed. The signal intensity at *m*/*z* 371 strongly depended on the disposable equipment used. When using plastic equipment, the intensity of this signal increased sharply. The *m*/*z* 371 signal is characteristic of bis(2-ethylhexyl) adipate, plasticizer (DEHA), or polyethylene glycol (PEG) [A_8_B + H]^+^ ((C_2_H_4_O)_n_H_2_O). However, no signals characteristic of amygdalin were observed. The MS^2^ fragmentation spectrum of *m*/*z* 371 signal showed two signals from the fragmentation ions, *m*/*z* 352 [371-19 (H3O)^+^]^+^ and *m*/*z* 265 [371-106 (CH_2_CH_2_)_2_H_2_O]^+^. Low-intensity *m*/*z* 371 signal was observed when only glass equipment was used in the sample preparation process. No signals characteristic of amygdalin were observed. In serum solution, amygdalin undergoes a very rapid hydrolysis reaction to acid, which gave the low-intensity *m*/*z* 371 signal. This compound was converted to *m*/*z* 313 at a higher temperature (at 340 °C). The MS^2^ fragmentation spectrum of *m*/*z* 313 showed signals from the fragmentation ions *m*/*z* 296, *m*/*z* 285, *m*/*z* 235, *m*/*z* 180, and *m*/*z* 145 characteristic for the structure distribution with mass *m*/*z* 313 (Figure 19).

**Figure 20:**
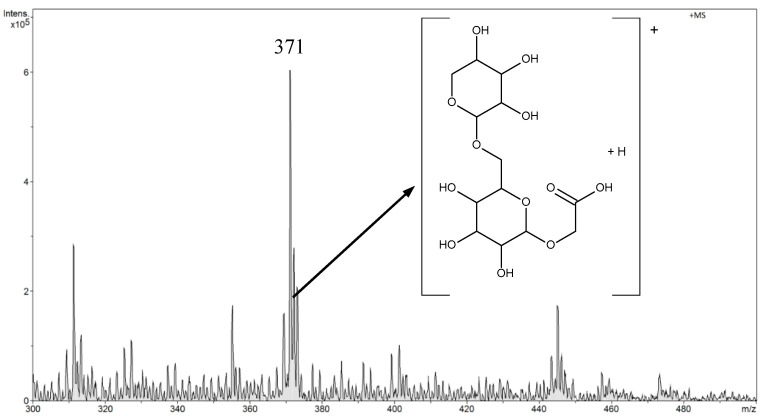
FAPA-MS spectrum of amygdalin in human serum at 200 °C.

**Figure 21:**
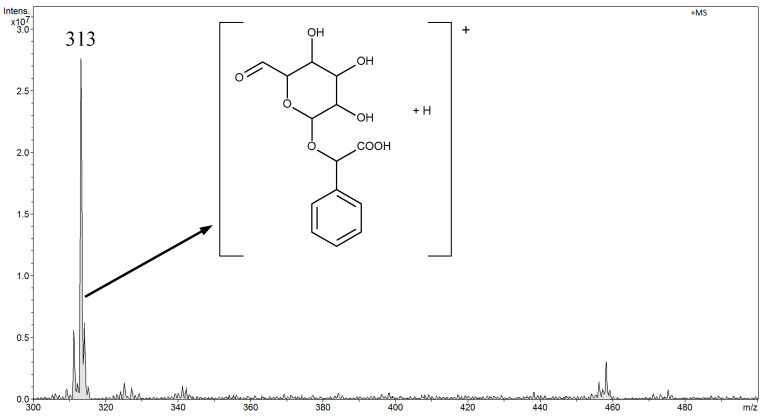
FAPA-MS spectrum of amygdalin in human serum at 340 °C.

The structures of both compounds are presented in Figure 20 and Figure 21. The signal compound *m*/*z* 313 is well-suited for identifying serum amygdalin concentration. Unfortunately, the detection threshold was relatively high and amounted to 0.1 mg of amygdalin in 1 mL of human serum in the FAPA-MS method.

Due to the rapid transformation of amygdalin in the human serum, it was impossible to pre-concentrate and separate amygdalin from the sample using Fe_3_O_4_@SiO_2_@VIN@MIP. The synthesized material showed selectivity toward amygdalin molecules, and the binding time of the analyte in the structure was up to 1 h. Therefore, it was impossible to bind amygdalin to Fe_3_O_4_@SiO_2_@VIN@MIP in the sample in a time shorter than the time during which the transformation of amygdalin in the biological environment takes place. It was not possible to lower the LOD for amygdalin in FAPA-MS by combining this method with the analyte pre-concentration technique using Fe_3_O_4_@SiO_2_@VIN@MIP.

## 4. Conclusions

This paper presented the methodology of direct determination of amygdalin in water, extracts, and biological materials using the ESI-MS and FAPA-MS methods. Both methods had a similar range of linearity, LOD, and LOQ. However, the FAPA-MS method was less sensitive to the presence of interferents in the analyzed sample. During the analysis of aqueous solutions of amygdalin with the addition of various metal cations, many of the complexes formed were observed in the ESI-MS spectrum. This was not recorded in the FAPA-MS method. Moreover, the FAPA-MS method was compared with the HPLC-UV/MS technique using a certified reference material. In order to pre-concentrate and isolate amygdalin from biological samples, the synthesis of Fe_3_O_4_@SiO_2_@MIP as molecular scavengers was proposed. They were used successfully to pre-concentrate amygdalin in aqueous and ethanol extracts. Moreover, the process of binding and releasing amygdalin from this material Fe_3_O_4_@SiO_2_@MIP was characterized. The aim of the research was the synthesis of amygdalin-selective Fe_3_O_4_@SiO_2_@MIP, which will enable the isolation and pre-concentration of the analyte from real samples. As a result, the analysis was to be characterized by better analytical parameters, and the material was to be used for the analysis of amygdalin using the FAPA-MS technique. The obtained sorption material could not be used for the pre-concentration and isolation of amygdalin from human serum. The mechanism of changes of amygdalin taking place in the human serum was proposed, as well as the structure of the resulting compounds, and analytical signals enabling the determination of amygdalin in this medium were selected.

The described methods allow for effective determination of amygdalin in aqueous solutions, environmental samples, and biological materials.

## Figures and Tables

**Table 1:** Analytical methods used in the analysis of amygdalin in a deferent matrix.

Materials	Analytical Methods	LOD	The Range of Measured Amygdalin Concentration (mg g^−1^)	References
**Different tissues of loquat fruit cv. “Dahongpao”**	Extraction and HPLC/UV	-	2.44–16.70	[20]
**Seeds of apples**	Solid-to-liquid extraction and HPLC/UV	0.0505 mg g^−1^	-	[17]
**Seeds, kernels, and food products**	Extraction and HPLC/UV	0.1 µg mL^−1^	0.01–17.50	[1]
**Apricot kernel**	Extraction and HPLC/UV	-	0.217–0.284	[21]
**Bitter almond products: raw, stir-fried, and scalded**	HPLC-ESI-MS^2^ and HPLC-DAD	2 µg mL^−1^	-	[12]
**Food extracts**	The enzyme immunoassay test	2.7 × 10^−7^ M	-	[22]
**Rat plasma**	Solid-phase extraction and LC-MS^2^	1.25 ng mL^−1^	-	[23]
**Rat plasma**	Extraction and UPLC	-	-	[24]
**Rat plasma**	HPLC	4.8 mg L^−1^	-	[25]
**Rat urine**	LC-MS^2^	-	-	[26]

**Table 2:** Results of amygdalin determination in aqueous solutions by ESI-MS and FAPA-MS methods.

Amygdalin Concentration (mg L^−1^)	Analysis of Amygdalin in Aqueous Solutions by ESI-MS			Analysis of Amygdalin in Aqueous Solutions by FAPA-MS		
	c_min_ (mg L^−1^)	c_max_ (mg L^−1^)	c_mean_ ± SD (mg L^−1^)	c_min_ (mg L^−1^)	c_max_ (mg L^−1^)	c_mean_ ± SD (mg L^−1^)
**45.0**	42.0	50.0	45.7 ± 1.4	38.0	52.0	44.7 ± 2.2
**0.45**	0.40	0.51	0.45 ± 0.02	0.39	0.52	0.46 ± 0.04
**0.0045**	0.0038	0.0054	0.0047 ± 0.0002	0.0036	0.0053	0.0045 ± 0.0002
**0.000045**	nd	nd	nd	nd	nd	nd

nd, not detected; SD, standard deviation.

**Table 3:** Summary of analytical parameters.

Analytical Parameter	Analytical Method	
	ESI-MS	FAPA-MS
**Linear range**	4.5 μg L^−1^–45 mg L^−1^	4.5 μg L^−1^–45 mg L^−1^
**LOD**	0.101 ± 0.003 μg L^−1^	0.050 ± 0.002 μg L^−1^
**LOQ**	0.303 ± 0.009 μg L^−1^	0.150 ± 0.006 μg L^−1^

**Table 4:** Results of amygdalin determination in apricot kernels (6.25 mg g^−1^) by HPLC-UV/MS and FAPA-MS methods.

Amygdalin Concentration (6.25 mg g^−1^)	HPLC-UV/MS			FAPA-MS Used Fe_3_O_4_@SiO_2_@VIN@MIP		
	c_min_ (mg g^−1^)	c_max_ (mg g^−1^)	c_mean_ ± SD (mg g^−1^)	c_min_ (mg g^−1^)	c_max_ (mg g^−1^)	c_mean_ ± SD (mg g^−1^)
**Water extract**	5.11	6.31	5.68 ± 0.21	6.22	6.67	6.46 ± 0.12
**Ethanol extract**	5.82	6.36	6.10 ± 0.17	6.45	6.81	6.63 ± 0.14

**Table 5:** Amygdalin adsorption capacity by Fe_3_O_4_@SiO_2_@VIN@MIP.

The Amount of Amygdalin in 5 mL Sample (μg)	The Amount of Amygdalin Remaining in the Aqueous Solution after Adding Fe_3_O_4_@SiO_2_@VIN@MIP (20 mg), Determined by ESI-MS			Amount of Adsorbed Amygdalin in Fe_3_O_4_@SiO_2_@VIN@MIP-Amygdalin (20 mg), Determined by FAPA-MS		
	amount_min_ (μg)	amount_max_ (μg)	amount_mean_ (μg)	amount_min_ (μg)	amount_max_ (μg)	amount_mean_ ± SD (μg)
225.0	nd	nd	nd	206.0	257.1	230.3 ± 7.2
2.25	nd	nd	nd	1.97	2.41	2.27 ± 0.11
0.0225	nd	nd	nd	0.0179	0.0299	0.0231 ± 0.0009
0.00022	nd	nd	nd	0.00015	0.00031	0.00024 ± 0.00002

nd, not detected; SD, standard deviation.

**Table 6:** Release of amygdalin from Fe_3_O_4_@SiO_2_@VIN@MIP-amygdalin in aqueous solution.

The Amount of Amygdalin in 100 mg Fe_3_O_4_@SiO_2_@VIN@MIP-Amygdalin (mg)	The Amount of Amygdalin in 10 mL Solution after Release from 100 mg Fe_3_O_4_@SiO_2_@VIN@MIP-Amygdalin, Determined by ESI-MS			Remaining Amygdalin in 100 mg Fe_3_O_4_@SiO_2_@VIN@MIP after the Release Process, Determined by FAPA-MS		
	amount_min_ (mg)	amount_max_ (mg)	amount_mean_ ± SD (mg)	amount_min_ (mg)	amount_max_ (mg)	amount_mean_ ± SD (mg)
7.0	6.74	7.50	6.9 ± 0.4	0.01	0.05	0.02 ± 0.02
5.0	4.85	5.23	5.04 ± 014	0.001	0.008	0.005 ± 0.003
2.0	1.92	2.14	2.02 ± 0.09	nd	nd	nd

nd, not detected; SD, standard deviation.

**Table 7:** The content of amygdalin in bitter almond kernels.

Materials	Amygdalin Content in Bitter Almond Kernels (mg/g), Determined by ESI-MS from the Extract			Amygdalin Content in Bitter Almond Kernels (mg/g), Determined by FAPA-MS from Fe_3_O_4_@SiO_2_@VIN@MIP-Amygdalin after Binding of the Analyte from the Extract		
	min.	max.	mean ± SD	min.	max.	mean ± SD
**Water extract**	19.9	26.1	22.1 ± 0.7	21.9	30.1	27.7 ± 1.2
**Ethanol extract**	34.8	44.5	37.9 ± 1.7	40.7	61.2	48.4 ± 1.8

SD, standard deviation.

**Table 8:** Analysis of amygdalin skin penetration.

Sample Number	Amount of Amygdalin per 1 cm^2^ of Skin after Release from Tape, Determined by ESI-MS (mg)		
	min.	max.	mean ± SD
**1**	26.119	31.211	28.783 ± 0.962
**2**	0.055	0.081	0.067 ± 0.004
**3**	0.004	0.005	0.0040 ± 0.0002
**4**	nd	nd	nd

nd, not detected; SD, standard deviation.

## Supplementary Materials

The following are available online at https://www.mdpi.com/2218-273X/10/10/1459/s1, Figure S1: XPS spectra obtained from Fe_3_O_4_@SiO_2_@VIN@MIP-amygdalin, Figure S2: XPS spectra obtained from Fe_3_O_4_@SiO_2_@VIN@MIP-amygdalin.
