# Supplementary material for: Analysis of Amygdalin in Various Matrices Using Electrospray Ionization and Flowing Atmospheric-Pressure Afterglow Mass Spectrometry
Source: Biomolecules. 2020 Oct 19;10(10):1459. doi: 10.3390/biom10101459 (PMC7603377; doi:10.3390/biom10101459)
Supplement: Supplementary file 1 [file biomolecules-10-01459-s001.pdf]

Supplementary Materials

Analysis of amygdalin in various matrices using the electrospray ionization and flowing atmospheric pressure afterglow mass spectrometry

Maria Guć [1]*, Sandra Rutecka [1] and Grzegorz Schroeder [1]

[1] Faculty of Chemistry, Adam Mickiewicz University in Poznań, Uniwersytetu Poznańskiego 8, 61-614 Poznań, Poland; maria.guc@amu.edu.pl, schroede@amu.edu.pl

* Correspondence: maria.guc@amu.edu.pl

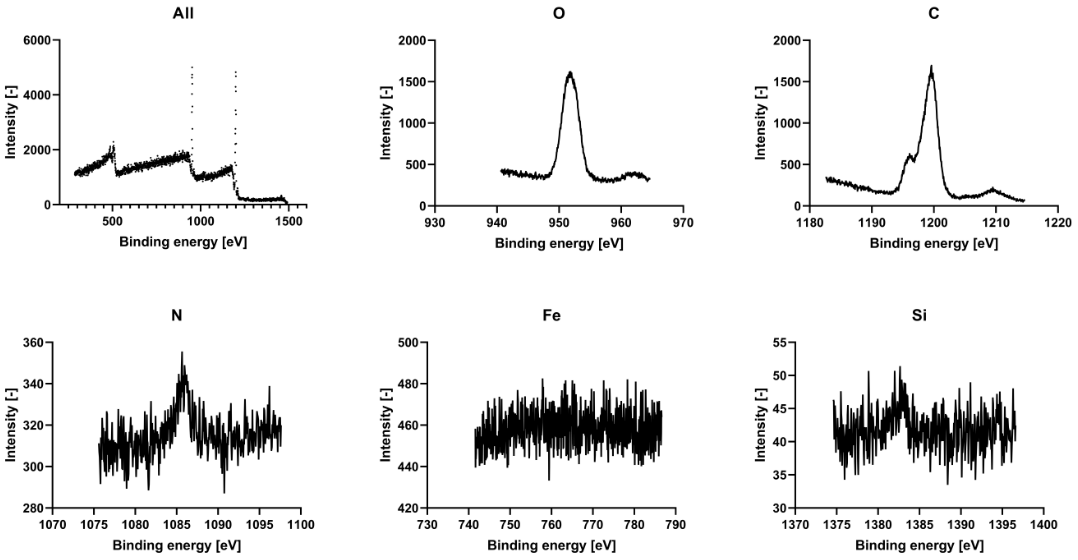

Figure S1. XPS spectra obtained from $Fe_3O_4@SiO_2@VIN@MIP$-amygdalin.

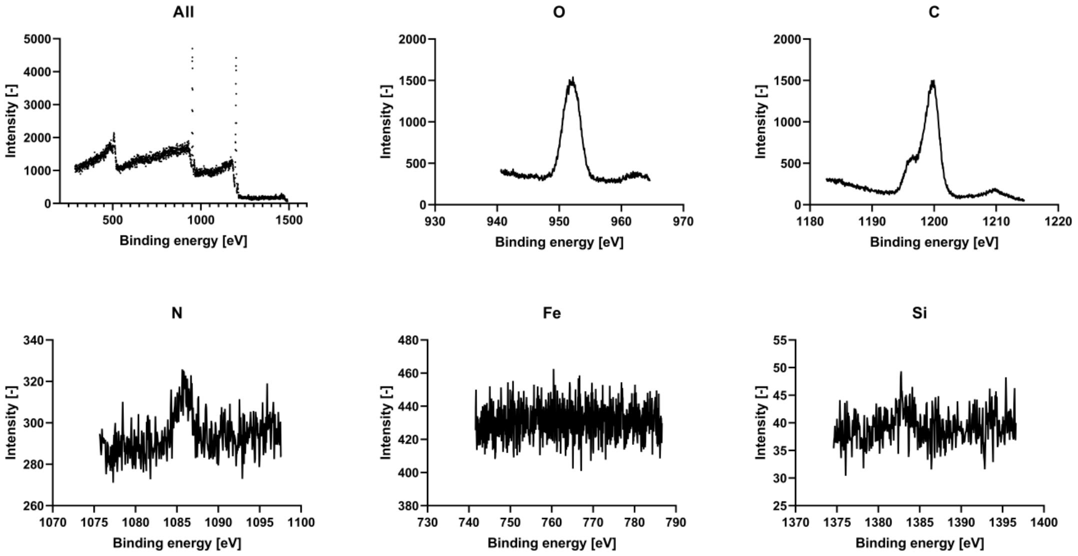

Figure S2. XPS spectra obtained from $Fe_3O_4@SiO_2@VIN@MIP$-amygdalin.
